# Case of Rapid Progression of Hemiatrophy on the Face: A New Clinical Entity?

**DOI:** 10.1155/2015/478640

**Published:** 2015-08-25

**Authors:** Hisashi Nomura, Shohei Egami, Tomoaki Yokoyama, Makoto Sugiura

**Affiliations:** Department of Dermatology, Shizuoka Municipal Shimizu Hospital, 1231 Miyakami, Shimizu-ku Shizuoka 424-0911, Japan

## Abstract

A lot of diseases, including lupus profundus, morphea, lipodystrophy, and Parry-Romberg syndrome, may manifest progressive hemifacial atrophy. These diseases usually progress slowly and rapid progression of atrophy is extremely rare. We report a case of elderly-onset rapid progression of hemifacial atrophy only in three weeks. Our case did not meet variable differential diagnoses. We discuss the clinical character of the patient against the past of literature and suppose it may be a new clinical entity.

## 1. Introduction

Various diseases, such as lupus profundus, morphea, lipodystrophy, and Parry-Romberg syndrome, may manifest progressive facial hemiatrophy. Atrophy in these diseases usually progresses slowly over months or years, and rapid progression of atrophy is extremely uncommon. We report a patient with late-onset (65 years old) rapid progression of hemifacial atrophy only in three weeks. Our patient does not meet various differential diagnoses. We discuss the clinical character of the patient against the past of literature and suspect it may be a new clinical entity.

## 2. Case Report

**Figure 1:**
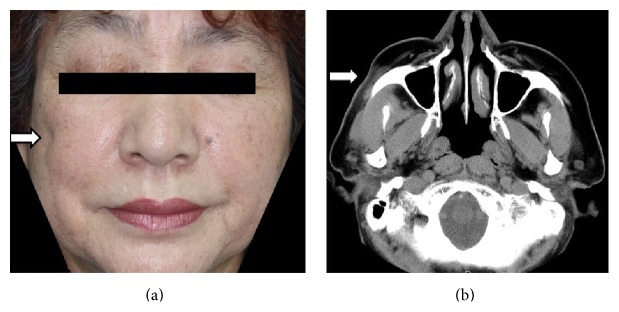
(a) Clinical manifestation at admission. (b) Axial computed tomography images, with soft-tissue settings, showing atrophy of subcutaneous fat on the right side of the face.

A 65-year-old woman was admitted to our department with complaints of facial asymmetry. The patient and her family noted that skin atrophy of the right cheek had progressed three weeks ago. Physical examination revealed soft-tissue atrophy of right cheek without any symptoms (Figure 1(a)). She was otherwise healthy and had not experienced any notable trauma or injection record at the lesion. There was no family history of craniofacial defects. The laboratory examination revealed slightly high levels of rheumatoid factor (RF) (26 IU/mL), but other inspection items, including antinuclear antibody and anti-double-strand DNA antibody, were within normal limits. Computed tomography scan revealed subcutaneous tissue atrophy at the right upper jaw (Figure 1(b)). The maxillary bone was not involved. The histopathology of the lesion showed the degeneration of adipocytes without inflammatory cell infiltration in fat layer and almost normal epidermis and connective tissues of dermis. Neurological disorder was not detected by neurological physical examination, electroencephalogram, and magnetic resonance imaging. She did not require any treatment. No progression of atrophy was observed one year later from the first admission at the time of this writing.

## 3. Discussion

Although variable diseases may cause progressive facial hemiatrophy, the clinical course and characteristic in our patient are definitely different from those of conventional disorder. Lupus profundus and morphea profundus were not consistent with histopathological and laboratory analysis results of our patient. The clinical course and normal cholesterol and glucose metabolism of the patient were inconsistent with lipodystrophy. Fat atrophy due to trauma or injection at the lesion was also ruled out by the patient. One of the other representative diseases which manifest facial hemiatrophy is Parry-Romberg syndrome (PRS) [1–3]. However, PRS usually begins in the first or second decade of life [4], and it progresses slowly over 2 to 20 years before stabilizing [1]. The clinical course of our patient is inconsistent with PRS. It is notable that, in our patient, the atrophy had progressed in only 3 weeks. However, Blair, in a review of 772 cases of progressive facial hemiatrophy, noted that “in some cases, it seems to ‘burn itself out' suddenly at a very early stage, resulting in only a minimal deformity, the atrophy usually limited to the distribution of one of the trigeminal nerve branches” [5]. This description closely matches the features of our patient. It may be possible that our cases and Blair's cases would be categorized as another subtype of hemifacial atrophy and a new clinical entity. We suppose that these cases have been overlooked because of inconsistency with conventional disorder and its minimal and subtle deformity. More cases should be studied to clarify the features of such a patient group.
